# Effect of Prehabilitation in Lung Cancer Patients Undergoing Lobectomy: A Review

**DOI:** 10.7759/cureus.49940

**Published:** 2023-12-05

**Authors:** Vaishnavi S Sharma, Vaishnavi Yadav

**Affiliations:** 1 Department of Cardiorespiratory Physiotherapy, Ravi Nair Physiotherapy College, Datta Meghe Institute of Higher Education & Research (DU), Wardha, Maharashtra, IND

**Keywords:** length of hospital stay, quality of life, 6mwt, lobectomy, lung cancer, prehabilitation

## Abstract

Deaths from lung cancer are mostly caused by smoking. Cough, dyspnea, fatigue, weight loss, and Horner's syndrome are among the symptoms. Non-small cell lung cancer (NSCLC) and small cell lung cancer (SCLC) are the two categories into which lung cancer may be divided. Because of its effectiveness and lower death rates, lobectomy is the primary line of therapy for benign and early-stage lung illnesses. Pulmonary rehabilitation is a routine treatment for thoracic surgery individuals who are at a high risk to improve functional ability, avoid postoperative deterioration, avoid postoperative deterioration, and reduce complications and even hospital stays. Pulmonary rehabilitation is a multifaceted continuum of services intended to help individuals with pulmonary disease and their families reach and sustain their highest possible degree of independence and community functioning, typically provided by an interdisciplinary team of specialists.

The objective of this research was to gather preliminary information and assess the effects of pre-rehabilitation on those suffering from lung cancer and having lobectomy. The pre-rehabilitation program's outcomes include increased lung functional capacity, enhanced quality of life, patient independence in daily living activities, and a shorter hospital stay. Gradually increasing walking distance over time can build endurance, requiring consistency, pacing, proper hydration, nutrition, and regular breaks. This review analyzed the effect of pre-rehabilitation in lung cancer patients undergoing lobectomy. Pre-rehabilitation program for individuals with lung cancer improves both preoperative and postoperative health through various exercises. Pulmonary rehabilitation is a multidisciplinary approach that encourages physical activity, learning about disease, treatment options, and coping mechanisms. Instead of curing the illness, its goal is to lessen its symptoms and limitations.

Patients with pulmonary diseases or undergoing thoracic surgery prefer pre-rehabilitation programs due to their non-traumatic nature and fewer resources required. Elastic resistance band exercises are beneficial for lung cancer patients' pre-rehabilitation by strengthening and stretching muscle groups, improving exercise capacity, and supporting white blood cell counts. These exercises can be customized to individual needs, making them a safe and effective addition to a patient's exercise routine. They have to be carried out at least three days a week. Pulmonary exercise, including the use of a tri-ball pulmonary exerciser or three-ball spirometer, can improve lung function, respiratory muscle strength, and exercise capacity in lung cancer patients. It involves breathing techniques, cough exercises, and inflating a balloon. Pulmonary rehabilitation has a positive impact on patient health. Improved lung vital capacity, shorter hospital stays, and fewer problems following surgery are all achieved with pulmonary rehabilitation. The pre-rehabilitation plan allows the patient to resume their daily routines.

## Introduction and background

Lung cancer is a highly frequent malignancy globally with notable fatality rates [1]. Bronchogenic carcinoma, another name for lung cancer, which originates in the bronchi or lung parenchyma, is an important factor in deaths from cancer in the United States (US). The primary cause of its sharp increase in subsequent decades is the rise in both male and female smoking, which was once uncommon [2]. The disease is often diagnosed late because it has no symptoms at first, which frequently results in a bad prognosis [3]. Smoking is linked to lung cancer in 90% of cases, with males being more at risk. The risk is increased by 20-30% by using other tobacco products, such as pipes and cigars. The risk is also raised by 20-30% by passive smoking. Around 90% of occurrences of lung cancer are related to smoking, with men being more susceptible. Using pipes and cigars, among other tobacco products, increases the risk by twenty to thirty percent. Passive smoking also raises the risk by twenty to thirty percent with other additional pollutants and metals and causes other lung disorders such as idiopathic pulmonary fibrosis [4]. Lung cancer represents 12.4% of all cases worldwide and is the leading cause of cancer-related mortality. It is also the most common disease worldwide. Even with better treatment, women's five-year survival rates were 24%, higher than men's, which were at 17%. The primary treatment modalities include surgery, radiation, and chemotherapy, with treatment selection dependent on the kind, stage, location, and health of the patient's tumour [5]. Physiotherapists should be aware of and manage symptoms, including cough, breathing difficulty, chest discomfort, exhaustion, appetite loss, and Horner's syndrome, characterized by a tiny pupil and drooping eyelid [6]. Lung cancer is categorized into two groups: non-small cell lung cancer (NSCLC) and small cell lung cancer (SCLC), with NSCLC accounting for 85% and SCLC accounting for 15%, respectively [7].

Lobectomy

Dr. Hugh Morriston Davies conducted a lobectomy in 1913, surgically excising the whole lung lobe. Incisions of less than 8 cm are commonly used to treat both benign and malignant conditions such as lung abscess, emphysema, fungal infection, and lung cancer [8]. Video-assisted thoracoscopic surgery (VATS) and robotic-assisted thoracoscopic surgery are effective methods for treating both benign and malignant lung diseases. However, a significant pulmonary reserve is necessary for a successful resection [9]. Risks associated with surgical procedures involving the pleural space and lungs include bleeding, accumulation of pus or fluid, lung collapse, bronchopleural fistula, empyema, infections, pneumothorax, haemorrhage, and other potential complications [10]. Due to growing evidence that VATS lobectomy reduces mortality and morbidity rates and is effective, it is presently advised as the initial treatment option in some cases of early-stage lung cancer and benign lung illnesses [11].

Prehabilitation

Thoracic surgery frequently uses pre-rehabilitation to enhance functional capacity and avoid postoperative deterioration, particularly for high-risk patients receiving lung transplants and volume reduction surgery [12]. Pulmonary rehabilitation is done in order to enhance the emotional and physical health of those suffering from long-term pulmonary conditions; this programme promotes sustained dedication to activities that improve health [13]. Over the last 10 years, research has demonstrated the many advantages of pulmonary rehabilitation for exercise training for people with chronic obstructive pulmonary disease (COPD). A crucial element in improving the effectiveness of rehabilitation for patients who have undergone lung transplants is comprehensive care tailored to their individual needs [14]. Pulmonary rehabilitation is a comprehensive surgery that seeks to reduce symptoms such as tiredness and dyspnea while improving quality of life, exercise tolerance, and functional status [15]. 

A multifaceted progression of services provided to individuals with pulmonary disease and their families, typically by an interdisciplinary team of specialists, with the goal of achieving and maintaining the individual's maximum level of independence and community functioning, is known as pulmonary rehabilitation [16]. Before surgery, pulmonary rehabilitation is an essential part of individualised, family-focused, multidisciplinary care. This entails medical professionals, nurses, respiratory therapists, physical therapists, occupational therapists, dietitians, and psychologists [17]. Pulmonary rehabilitation, involving interventional techniques and exercise, has been shown to reduce hospital stays and postoperative issues in patients undergoing lobectomies or lung resections [18]. For two to four weeks, patients in the pulmonary rehabilitation group had training in peripheral muscle training, respiratory exercise, and appropriate breathing methods under the supervision of physiotherapists. Exercises for peripheral circulation, aerosol treatment, mobilising the shoulder girdle, and expanding the chest were all part of the pulmonary rehabilitation that was started [19]. It is possible to decrease atelectasis, avoid hospital-acquired infections, and speed up recovery from major thoracic surgery by combining preoperative oxygen use with a six-minute walk distance [20].

## Review

Methodology

**Figure 1:**
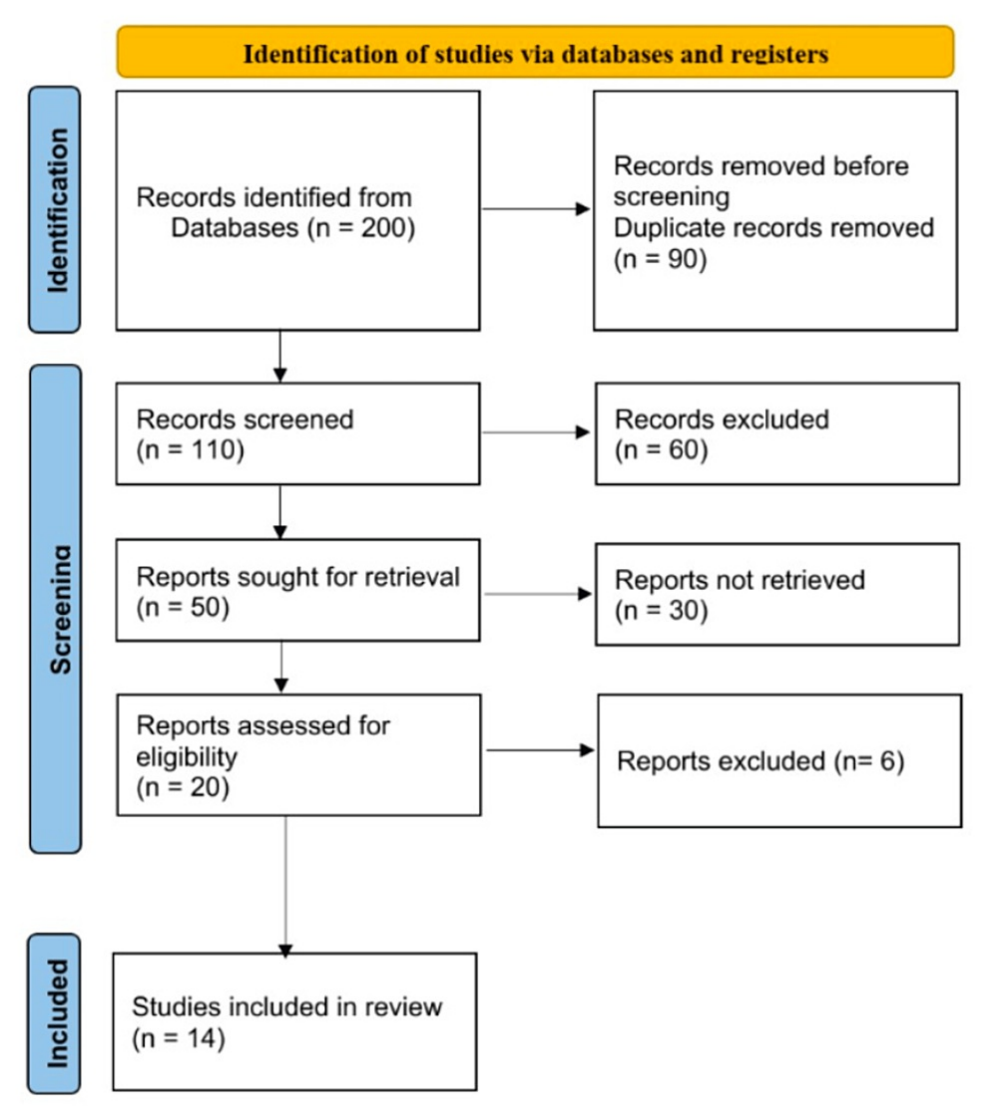
Preferred Reporting Items for Systematic Reviews and Meta-Analyses flowchart.

Articles were searched on databases like Google Scholar and PubMed with the keywords lung cancer, lobectomy, prehabilitation, pulmonary rehabilitation, peak oxygen uptake (Vo2 peak), six-minute walk test, health-related quality of life, SCLC, NSCLC stages (I-IV), lung surgery, and physical manipulation pulmonary rehabilitation. Relevant publications were evaluated and included in this review, as shown in Table 1. Academic publications, original research, case-control studies, randomised trials, and cross-sectional studies were all taken into consideration. The review study analyses the effect of prehabilitation in lung cancer patients undergoing lobectomy by rating and classifying inclusion criteria by subjects who were willing to participate in studies, both male and female, including smokers, participants diagnosed with lung cancer, lobectomy, and acute condition, and exclusion criteria by participants with nerve involvement, rib fracture, recent implantation, and recent coronary artery bypass graft. After manually searching and eliminating unwanted and duplicate articles, 15 articles were selected for this review article (Figure 1), which is shown in the PRISMA flow diagram.

**Table 1:** A summary of the articles reviewed for LC and lobectomy patients. LC: Lung cancer; 6MWT: Six minutes walk test; PFT: Pulmonary function test; CVPR: Conventional pulmonary rehabilitation; PMPR: Physical manipulation pulmonary rehabilitation; VO2 max: Maximum oxygen uptake; VO2 peak: Peak oxygen uptake; NSCLC: Non-small cell lung cancer; SCLC: Small cell lung cancer; CRT: Conventional resistance training; WBV: Whole body vibration; ADLs: Activities of daily living; HRQOL: Health-related quality of life; QOL: Quality of life; LOS: Length of stay; WBVT: Whole-body vibration training; NIV: Non-invasive ventilation; VATS: Video-assisted thoracoscopic surgery

Sr. no	Author and year of publication	Study type and sample size	Outcome measure	Intervention	Result	Finding
1	Sahin et al. 2022 [21].	Prospective study with 66 LC patients	6MWT	The program involved patients participating in two-hour weekly sessions for eight weeks, incorporating breathing, relaxation, stretching, peripheral muscle strengthening, and aerobic exercises.	Increase in six-minute walking distance.	After lung resection, exercise ability can be enhanced via pulmonary rehabilitation. There are considerably lower dyspnea symptoms, enhancing the standard of living for those suffering from NSCLC.
2	Zhou et al. 2022 [22].	Randomized controlled trial with 86 LC patients	PFT, 6MWT	An examination of 86 patients divided into two groups: one received CVPR and PMPR for 21 days while the control group received only CVPR.	The peak expiratory flow was higher (316 ± 95 vs. 272 ± 103 l/min, respectively) in comparison to the control group (p = 0.043).	Pulmonary manipulation and pulmonary rehabilitation are methods that enhance lung functional capacity and prevent post-operative complications.
3	Liu et al. 2020 [23].	Randomized controlled trial with 73 patients	6MWT	Before surgery, patients underwent two weeks of multimodal treatments, including jogging, walking, or cycling, resistance exercises, and pulmonary exercise using a tri-ball pulmonary exerciser, breathing techniques, and balloon inflating.	Average 6MWT was 60.9 minutes higher preoperatively in the prehabilitation group.	For patients having a VATS lobectomy for LC, a two-week home-based multimodal prehabilitation programme may greatly enhance postoperative functional capacity.
4	Messaggi – sartor et al. 2019 [24].	Pilot randomized controlled trial with 37 LC patients	Vo2 peak	The training program included aerobic exercises and high-intensity respiratory muscle training.	VO2 peak improved considerably (2.13 mL/Kg/min (95% CI 0.06 to 4.20)) during the eight-week training programme.	An eight-week program involving high-intensity respiratory muscle training and aerobic exercise significantly improves exercise capacity and respiratory muscle strength.
5	Boujibar et al. 2018 [25].	Retrospective cohort study with 38 LC patients	Vo2 peak	The prehabilitation program involved exercise retraining, muscular strengthening, therapeutic education, and smoking cessation, with weekly sessions lasting 90 minutes until the operating date.	Changes in VO2 peak.	Prehabilitation significantly reduces the occurrence and severity of postoperative complications following minimally invasive pulmonary lobectomy surgery.
6	Cavalheri et al. 2017 [26].	Pilot randomized controlled trial with 17 NSCLC (I–IIIA) patients	Exercise capacity (VO2 peak and 6MWT)	Supervised individual muscle strength and aerobic program for eight weeks vs. control group	Changes in VO2 peak and 6MWT	The exercise group showed significant improvements in the peak rate of oxygen consumption.
7	Salhi et al. 2015 [27].	Randomized controlled trial with 70 NSCLC (I–IV), SCLC (LD), and mesothelioma (I–III) patients	6MWT	12 weeks of CRT vs. WBV vs. usual care	The study investigates the impact of a training program on the strength, quality of life, and maximal capacity of 6MWT quadriceps force in CRT.	LC patients' exercise ability is significantly impacted by radiation therapy, with WBVT partially replacing CRT, but CRT significantly improves and recovers functional exercise ability.
8	Bagan et al. 2013 [28].	Prospective study with 20 LC patients	Vo2 max	The protocol included a cardiorespiratory rehabilitation program and three hours of noninvasive ventilation, followed by three weeks of therapy and repeated functional tests.	VO2 max increase of 12%	Preoperative NIV in pulmonary rehabilitation allows surgery for patients not initially eligible for resection, necessitating evaluation of long-term outcomes survival compared to non-surgical therapies.
9	Stigt et al. 2013 [29].	Controlled, randomised study with 57 NSCLC patients	6MWT	Compared to standard care, an aerobic program lasting 12 weeks is followed by three and then six months of follow-up.	Six-minute walking distance improved by 35 minutes from the preoperative baseline	The 6MWD test showed an 8% improvement after two months of exercise training, and 43% and 32% improvements after a rehabilitation program.
10	Divisi et al. 2013 [30].	Prospective study with 27 LC patients	VO2 max	The daily PPR consisted of 50 minutes of respiratory physiotherapy and 40 minutes of aerobic work, including 20 minutes of cycling and walking.	Nine patients completed rehabilitation treatment, with VO2 max increasing significantly. Patients underwent lobectomy, with a 15% postoperative morbidity rate.	Pulmonary rehabilitation has a positive impact on patient health; it improves lung vital capacity.
11	Quist et al. 2012 [31].	Single-arm trial with 29 NSCLC stage (III–IV) patients	VO2 peak, QOL, and 6MWT	Exercise program for muscle strength and aerobics.	The study found significant increases in muscle strength measurements, including VO2 peak and six-minute walk distance, with a p-value of less than 0.05.	The study showed significant improvements in VO2 peak, muscle strength, emotional well-being, and six-minute walk distance, but no significant changes in HRQOL.
12	Arbane et al. 2011 [32].	Randomized controlled trial with 53 NSCLC patients	QOL, 6MWT	The study compares the effectiveness of an aerobic exercise program vs. usual care for a 12-week strength program.	Stay time and surgical complications for 6MWT power.	Strength declined in the control group.
13	Benzo et al. 2011 [33].	Randomized prospective with 10 LC patients	QOL, LOS	Breathing exercise, strengthening and stretching exercise, and aerobic exercise.	Exercise improved breathing and quality of life, making patients independent in ADLs; there was a decrease in the length of hospital stay.	Pulmonary rehabilitation reduces the length of hospital stay and decreases post-operative complications.
14	Cesario et al. 2007 [34].	Pilot study with eight LC patients	6MWT	The patient underwent supervised incremental exercise until they completed 30 minutes of continuous cycling at 70-80% of their maximal work.	Increased distance in six-minute walk test (6MWT)	The study suggests that early rehabilitative intervention can prevent deterioration and accelerate function recovery in surgical patients resected for NSCLC, leading to improved exercise tolerance.

Discussion

Lung cancer, a major cause of cancer-related mortality in the United States, is primarily caused by increased smoking rates among both men and women [2]. The disease is frequently diagnosed in its early asymptomatic stage; however, it commonly progresses without symptoms until later stages, leading to a poor prognosis [3]. Smoking is responsible for 90% of lung cancer cases with men at higher risk. Passive smoking increases risk by 20-30%. Factors like toxins, metals, and lung conditions also increase risk [4]. Physiotherapists must possess proficiency in addressing a range of symptoms associated with the illness, including chest discomfort, fatigue, coughing, dyspnea, appetite loss, and Horner's syndrome [6]. NSCLC patients generally have higher survival rates compared to SCLC patients [7]. A lobectomy, which treats both benign and malignant lung illnesses, involves surgically removing the whole lung lobe [9]. Pleural space lung surgical procedures have many potential dangers; these include infection, pneumothorax, haemorrhage, bronchopleural fistula, empyema, and pleural effusion. In addition to bleeding, bronchopleural fistula, pus in the chest cavity, and fluid in the lungs and chest wall, these dangers also include lung collapse [10]. Due to its efficiency in lowering mortality and morbidity rates, VATS lobectomy is advised as the main therapy for early-stage lung cancer and several benign lung illnesses [11]. 

A comprehensive approach called pulmonary rehabilitation aims to improve the physical and emotional well-being of people with chronic lung disorders and promote long-term health-enhancing practices. [13]. By promoting physical exercise and educating patients about their condition, available treatments, and coping techniques, rehabilitation strives to return patients to their highest degree of independent function [35]. Studies show pulmonary rehabilitation positively impacts lung cancer patients before lobectomy and post-resection, reducing dyspnea psychological symptoms, improving exercise ability, and improving the standard of living [21]. Following minimally invasive pulmonary lobectomy surgery, prehabilitation considerably lowers the incidence and severity of post-operative problems [25]. The workout group showed higher increases in the peak oxygen consumption rate [26]. The 6MWD test showed an 8% improvement after two months of exercise training and 43% and 32% improvements after a rehabilitation program [29]. Pulmonary rehabilitation shows the impact on patient health. It improves lung vital capacity [30]. Pulmonary rehabilitation reduces the duration of hospital stay and reduces problems following surgery [33].

## Conclusions

This review analyzed the impact of prehabilitation on patients with lung cancer who are undergoing lobectomy. The lung cancer patients' prehabilitation program improves preoperative and post-operative health through various exercises. Pulmonary rehabilitation is a multidisciplinary approach that encourages physical activity, learning about disease, treatment options, and coping mechanisms. It aims to reduce symptoms and disability from the disease rather than reversing it. Patients with pulmonary diseases or undergoing thoracic surgery prefer prehabilitation programs due to their non-traumatic nature and fewer resources required. Resistance exercises, including stretching and strengthening routines utilizing an elastic resistance band, need to be carried out thrice a week at a minimum. Pulmonary exercise (using a tri-ball pulmonary exerciser, breathing techniques, cough exercises, and inflating a tiny balloon). Pulmonary rehabilitation has a positive impact on patient health. It improves lung vital capacity, reduces hospital stay, and decreases post-operative complications. With the help of a rehabilitation program, patients easily get back to the activities of daily living.
